# Tom Lawry. Hacking Healthcare: How AI and the Intelligence Revolution Will Reboot an Ailing System: Routledge. Taylor & Francis Group, A Productivity Press Book: 376 pages; hard copy. ISBN: 988-1-032-26016-7

**DOI:** 10.3325/cmj.2022.63.402

**Published:** 2022-08

**Authors:** Stjepan Orešković

Are we ready for the complex physiological, social, economic, and cultural ramifications of living in the digital age? How can artificial intelligence (AI) be integrated into today's health systems with other technologies to transform health for patients?

**Content:** This 376-page book is dedicated to “those who believe health and medicine are noble causes and strive to make them so. The book shows how complex relations and demanding AI solutions may transform healthcare by employing techno-creations that have already reached human parity – “speech, vision, knowledge extraction and other capabilities previously unique to the human brain.” The book consists of 21 chapters. Each chapter begins with a brief introduction, followed by supporting figures, and ends with a list of relevant references. The first twelve chapters cover the most critical developments in the health care application of AI, machine learning, internet of things, robotics, and telehealth, mainly focusing on the intelligent health care revolution. There are numerous well-described examples of AI application to personalized medicine, opioid pandemic, mental health, chronic diseases, and aging. The second part focuses on creating a new data culture, data governance, social determinants of health, fairness, inclusiveness, transparency, accountability, privacy, and security. The third part answers the question of how AI-driven leadership and management should function, and lists the characteristics of AI leaders (strategically ambidextrous, customer-obsessed, agile, internal evangelist) and laggards (“wait and see guys,” treating data mainly as a by-product of existing activities). It ends with a chapter on the bond between cloud computing and AI (cloud is how you do computing, not where) and how that bond helped Moderna to deliver the first clinical batch of its vaccine candidate to the National Institute of Health for the first phase of clinical trials in less than 42 days. The last chapter, *The Future Is Not What It Used To Be*, starts with a quotation from Arthur Clarke, film director and author of *2001: A Space Odyssey*: “Any sufficiently advanced technology is indistinguishable from magic.”, listing the trends that will create the magic of the future: precision medicine, a quantum leap of health, 3D bioprinting, smart dust, emotional AI, biohacking, augmented and mixed reality, brain-computer interfaces, conversational AI.

Lawry analyzes, in a simple and consumable manner (the unique perspective that comes with the position of the National Director for Al, Health and Life Sciences at Microsoft), the strategies for digital transformation applied to performance optimization, including AI, machine learning, and cognitive services. This is the first time in the history of science that we are faced with a challenge to integrate all existing sources of data and information to confront a single phenomenon: the COVID-19 pandemic. What have we learned from fighting a global pandemic and how to apply these lessons to solve numerous health care challenges? By hacking the health care system? For Lawry, hacking is not a negative term. Hacking means understanding and addressing problems in a free manner to solve “the unsolvable.” Through teaching and empowering clinicians, patients, and other consumers of health care services to take control of what is essential and create a sustainable and intelligent health system, AI should function as a responsible intelligence, capable of doing good while used with respect, inclusion, and transparency. Timely access to relevant information is the key to transformation.

**Target audience:** This book is an excellent read for health care professionals interested in new technologies and their application to medicine and health care.

**Highlights:** In Hacking Healthcare, Tom Lawry describes the origins of Al, how Al is being used today, and what has to be done to make AI an indispensable part of health care delivery.

**Limitations:** The book highlights the most critical and dominating trends in AI application to health care and is therefore intended for readers acquainted with the basic principles of AI. The preferable format is hard copy because some illustrations cannot be easily viewed in the Kindle version.

The end of each chapter offers a list of references suitable for further reading, among them the following: Eric Topol Deep Medicine: How Artificial Intelligence Can Make Healthcare Human Again. Basic Books, New York, 2019. ISBN: 9781541644618 and Fred Trotter & David Uhlman Hacking Healthcare: A Guide to Standards, Workflows, and Meaningful Use. O'Reilly, 2013. ISBN: 9781449305024

**Commentary:** The book is a must-read for medical doctors, public health specialists, doctoral students, IT experts, economists in health care, and all practitioners who seek to comprehensively understand how to create and employ new technologies and create value-based health care. You will find this book insightful, whether you are an AI novice or a health care Al expert.

